# Supplementary material for: Space-time risk cluster of visceral leishmaniasis in Brazilian endemic region with high social vulnerability: An ecological time series study
Source: PLoS Negl Trop Dis. 2021 Jan 19;15(1):e0009006. doi: 10.1371/journal.pntd.0009006 (PMC7846114; doi:10.1371/journal.pntd.0009006)
Supplement: S1 Table — (DOCX) [file pntd.0009006.s002.docx]

**S2 Table 1. Components of SVI-UI dimension.**

| **Indicator** | **Description** | **Weight** |
| --- | --- | --- |
| a) Percentage of people in households with inadequate water supply and sanitation | Ratio between the number of people living in households whose water supply does not come from the general system and whose sanitation is not performed by sewage or septic tank, and the total population residing in permanent private housing, multiplied by 100. Only permanent private households are considered. | 0.3 |
| b) Percentage of population living in urban households without garbage collection service | Ratio between the population living in households without garbage collection and the total population living in permanent private housing units, multiplied by 100. Situations in which garbage collection is performed directly by a public or private company, or garbage is disposed in a bucket, tank, or warehouse away from home for later collection by the service provider are included. Only permanent private households located in urban areas are considered. | 0.3 |
| c) Percentage of people living in households with a per capita income of less than half a minimum wage and spending more than an hour to work in the total number of employed, vulnerable people returning from work daily. | Ratio between the number of employed persons of 10 years or older, living in households with per capita income less than half the minimum wage, August 2010, and who spend more than an hour to travel to the workplace, and the total number of employed persons in this age group living in households with per capita income below half the minimum wage, August 2010, and returning from work daily, multiplied by 100. | 0.3 |

**S2 Table 2. Components of SVI-HC dimension.**

| **Indicator** | **Description** | **Weight** |
| --- | --- | --- |
| a) Mortality up to 1 year old | Number of children who are not expected to survive their first year of life in every 1,000 live births. | 0.125 |
| b) Percentage of children 0-5 years old not attending school | Ratio between the number of children 0-5 years old not attending day care or school, and the total number of children in this age group (multiplied by 100) | 0.125 |
| c) Percentage of people aged 6 to 14 years of age not attending school | Ratio between the number of people aged 6 to 14 who do not attend school, and the total number of people in this age group (multiplied by 100). | 0.125 |
| d) Percentage of women 10 up to 17 years of age who had children | Ratio between the number of women 10 to 17 years old who had children, and the total number of women in this age group (multiplied by 100) | 0.125 |
| e) Percentage of female heads of household, without complete elementary school and with at least one child under 15 years of age | Ratio between the number of women who are household heads, who do not have completed elementary school, and who have at least one child under the age of 15 living in the household, and the total number of women household heads (multiplied by 100). Only permanent private households are considered. | 0.125 |
| f) Illiteracy rate of population ≥15 years | Ratio between the population aged 15 and over who cannot read or write a single ticket, and the total number of people in this age group (multiplied by 100). | 0.125 |
| g) Percentage of children living in households where none of the residents have completed elementary school | Ratio between the number of children under 14 living in households where none of the residents have completed elementary school, and the total population in this age group residing in permanent private housing (multiplied by 100). | 0.125 |
| h) Percentage of people aged 15 to 24 who do not study, do not work and have per capita household income equal to or less than half the minimum wage (2010), the total population in this age group | Ratio between 15-24 years old who do not study, do not work and whose per capita income is less than half the minimum wage, as of August 2010, and the total population in this age group (multiplied by 100). Only permanent private households are considered. | 0.125 |

**S2 Table 3. Components of SVI-I/W dimension.**

| **Indicator** | **Description** | **Weight** |
| --- | --- | --- |
| a) Proportion of persons with per capita household income equal to or less than half the minimum wage (2010) | Proportion of individuals with per capita household income equal to or less than R$ 255.00 monthly (in Reais in August 2010), equivalent to half the minimum wage on that date. The universe of individuals is limited to those living in permanent private households. | 0.2 |
| b) Population unemployment rate of 18 years and over | Percentage of economically active population (PEA) in this age group who were unemployed, that is, not employed in the week prior to the census date, but had sought work throughout the month preceding the date of this survey. | 0.2 |
| c) Men ≥ 18 years and over not attending school | Ratio between persons 18 years of age and over without elementary school, in informal occupation, and total population in this age group, multiplied by 100.  Informal occupation means that they work, but are not: employees with a formal contract, military, army, navy, aeronautics, military or fire brigade employees employed by the legal regime of civil servants or self-employed and self-employed workers. with contribution to official welfare institute. | 0.2 |
| d) Percentage of people in households with per capita income under half the minimum wage (in 2010) | Ratio of persons living in households with a per capita income below half a minimum wage, as of August 2010, in which the income of residents 65 years of age and over (older people) corresponds to more than half of total household income , and the total population residing in permanent private housing units (multiplied by 100). | 0.2 |
| e) Activity rate of persons 10 up to 14 years of age | Ratio of persons 10 to 14 years of age who were economically active, i.e. who were employed or unemployed in the census reference week among the total number of people in this age group (multiplied by 100). Unemployed means a person who, not being employed in the reference week, had sought work in the month prior to this survey. | 0.2 |
