# Supplementary material for: Space-time risk cluster of visceral leishmaniasis in Brazilian endemic region with high social vulnerability: An ecological time series study
Source: PLoS Negl Trop Dis. 2021 Jan 19;15(1):e0009006. doi: 10.1371/journal.pntd.0009006 (PMC7846114; doi:10.1371/journal.pntd.0009006)
Supplement: S2 Table — (DOCX) [file pntd.0009006.s003.docx]

| **Variables** | **AL** | **BA** | **CE** | **MA** | **PB** | **PE** | **PI** | **RN** | **SE** |
| --- | --- | --- | --- | --- | --- | --- | --- | --- | --- |
| **n(%)** | **(n = 1,227)** | **(n = 6,589)** | **(n = 7,953)** | **(n = 10,534)** | **(n = 740)** | **(n = 2,366)** | **(n = 4,289)** | **(n = 1,791)** | **(n = 1,009)** |
| **Case type** |  |  |  |  |  |  |  |  |  |
| New cases | 1,109 (90.38) | 5,918 (89.82) | 7,001 (88.03) | 9,816 (93.18) | 644 (87.03) | 2,092 (88.42) | 3,746 (87.34) | 1,629 (90.95) | 907 (89.9) |
| Relapse | 17 (1.39) | 282 (4.28) | 304 (3.82) | 341 (3.24) | 33 (4.46) | 74 (3.13) | 253 (5.9) | 58 (3.24) | 42 (4.16) |
| Transference | 4 (0.33 | 86 (1.3) | 183 (2.3) | 118 (1.12) | 6 (0.81) | 18 (0.76) | 23 (0.54) | 4 (0.22) | 5 (0.49) |
| Miss data | 97 (7.9) | 303 (4.6) | 465 (5.85) | 259 (2.46) | 57 (7.7) | 182 (7.69) | 267 (6.22) | 100 (5.58) | 55 (5.45) |
| **Sex** |  |  |  |  |  |  |  |  |  |
| Male | 742 (60.47) | 3,961 (60.12) | 5,189 (65.25) | 6,434 (61.08) | 459 (62.03) | 1,447 (61.16) | 2,781 (64.84) | 1,206 (67.34) | 669 (66.3) |
| Female | 484 (39.45) | 2,623 (39.81) | 2,759 (34.69) | 4,098 (38.9) | 279 (37.7) | 914 (38.63) | 1,507 (35.14) | 583 (32.55) | 340 (33.7) |
| Miss data | 1 (0.08) | 5 (0.07) | 5 (0.06) | 2 (0.02) | 2 (0.27) | 5 (0.21) | 1 (0.02) | 2 (0.11) | - |
| **Age** |  |  |  |  |  |  |  |  |  |
| 0-4 years | 500 (40.75) | 2,488 (37.76) | 2,706 (34.03) | 5,344 (50.73) | 251 (33.92) | 937 (39.6) | 1,841 (42.92) | 531 (29.65) | 282 (27.95) |
| 5-19 years | 440 (35.86) | 1,889 (28.67) | 1,575 (19.8) | 2,104 (19.97) | 177 (23.92) | 635 (26.84) | 724 (16.88) | 392 (21.89) | 275 (27.25) |
| 20-39 years | 215 (17.52) | 1,231 (18.68) | 1,834 (23.06) | 1,879 (17.84) | 184 (24.86) | 460 (19.44) | 979 (22.83) | 502 (28.03) | 255 (25.27) |
| 40-59 years | 53 (4.32) | 716 (10.87) | 1,291 (16.23) | 893 (8.48) | 95 (12.84) | 235 (9.93) | 529 (12.33) | 290 (16.19) | 150 (14.87) |
| ≥ 60 years | 18 (1.47) | 262 (3.98) | 542 (6.82) | 308 (2.92) | 33 (4.46) | 99 (4.18) | 216 (5.04) | 75 (4.19) | 46 (4.56) |
| Miss data | 1 (0.08) | 3 (0.04) | 5 (0.06) | 6 (0.06) | - | - | - | 1 (0.05) | 1 (0.1) |
| **Ethinicity /skin colour** | |  |  |  |  |  |  |  |  |
| White | 66 (5.38) | 779 (11.82) | 659 (8.29) | 950 (9.02) | 177 (23.92) | 225 (9.51) | 200 (4.66) | 279 (15.58) | 111 (11) |
| Non white | 644 (52.49) | 4,209 (63.88) | 5,809 (73.04) | 7,973 (75.69) | 378 (51.08) | 1,247 (52.7) | 3,579 (83.45) | 923 (51.53) | 696 (68.98) |
| Miss data | 517 (42.13) | 1,601 (24.3) | 1,485 (18.67) | 1,611 (15.29) | 185 (25) | 894 (37.79) | 510 (11.89) | 589 (32.89) | 202 (20.02) |
| **Zone** |  |  |  |  |  |  |  |  |  |
| Urban | 327 (26.65) | 3,229 (49) | 5,665 (71.23) | 7,320 (69.49) | 395 (53.38) | 954 (40.32) | 3,055 (71.23) | 1,271 (70.97) | 624 (61.84) |
| Rural | 849 (69.19) | 2,990 (45.38) | 2,029 (25.51) | 2,789 (26.47) | 319 (43.11) | 1,302 (55.03) | 1,102 (25.69) | 475 (26.52) | 335 (33.2) |
| Periurban | 33 (2.69) | 67 (1.02) | 51 (0.64) | 142 (1.35) | 1 (0,13) | 29 (1.23) | 21 (0.49) | 24 (1.34) | 32 (3.17) |
| Miss data | 18 (1.47) | 303 (4.6) | 208 (2.62) | 283 (2.69) | 25 (3.38) | 81 (3.42) | 111 (2.59) | 21 (1.17) | 18 (1.78) |
| **Level of education** | |  |  |  |  |  |  |  |  |
| < 8 years | 317 (25.84) | 2,002 (30.38) | 1,818 (22.86) | 2,704 (25.67) | 234 (31.62) | 698 (29.5) | 1,239 (28.89) | 442 (24.68) | 405 (40.14) |
| ≥ 8 years | 40 (3.26) | 384 (5.83) | 665 (8.36) | 860 (8.16) | 66 (8.92) | 120 (5.07) | 606 (14.13) | 184 (10.27) | 141 (13.97) |
| Miss data/N.A. | 870 (70.9) | 4,203 (63.79) | 5,470 (68.78) | 6,970 (66.17) | 440 (59.46) | 1,548 (65.43) | 2,444 (56.98) | 1,165 (65.05) | 463 (45.89) |
| **Outcome** |  |  |  |  |  |  |  |  |  |
| Cure | 674 (54.93) | 4,938 (74.94) | 6,043 (75.98) | 7,085 (67.25) | 453 (61.22) | 1,895 (80.09) | 2,626 (61.23) | 1,427 (79.68) | 829 (82.16) |
| Abandonment | 3 (0.24) | 21 (0.32) | 36 (0.45) | 43 (0.41) | 5 (0.68) | 5 (0.21) | 8 (0.19) | 10 (0.56) | 13 (1.29) |
| Death | 88 (7.17) | 501 (7.6) | 601 (7.56) | 700 (6.65) | 70 (9.46) | 209 (8.83) | 282 (6.57) | 127 (7.09) | 112 (11.1) |
| Transference | 39 (3.18) | 220 (3.34) | 446 (5.61) | 892 (8.47) | 26 (3.51) | 59 (2.5) | 96 (2.24) | 61 (3.4) | 10 (0.99) |
| Miss data | 423 (34.47) | 909 (13.8) | 827 (10.4) | 1,814 (17.22) | 186 (25.13) | 198 (8.37) | 1,277 (29.77) | 166 (9.27) | 45 (4.46) |

**Table 1. Baseline characteristics per state.**

N.A. not applicable
