# Supplementary figures and images for: Space-time risk cluster of visceral leishmaniasis in Brazilian endemic region with high social vulnerability: An ecological time series study
Source: PLoS Negl Trop Dis. 2021 Jan 19;15(1):e0009006. doi: 10.1371/journal.pntd.0009006 (PMC7846114; doi:10.1371/journal.pntd.0009006)

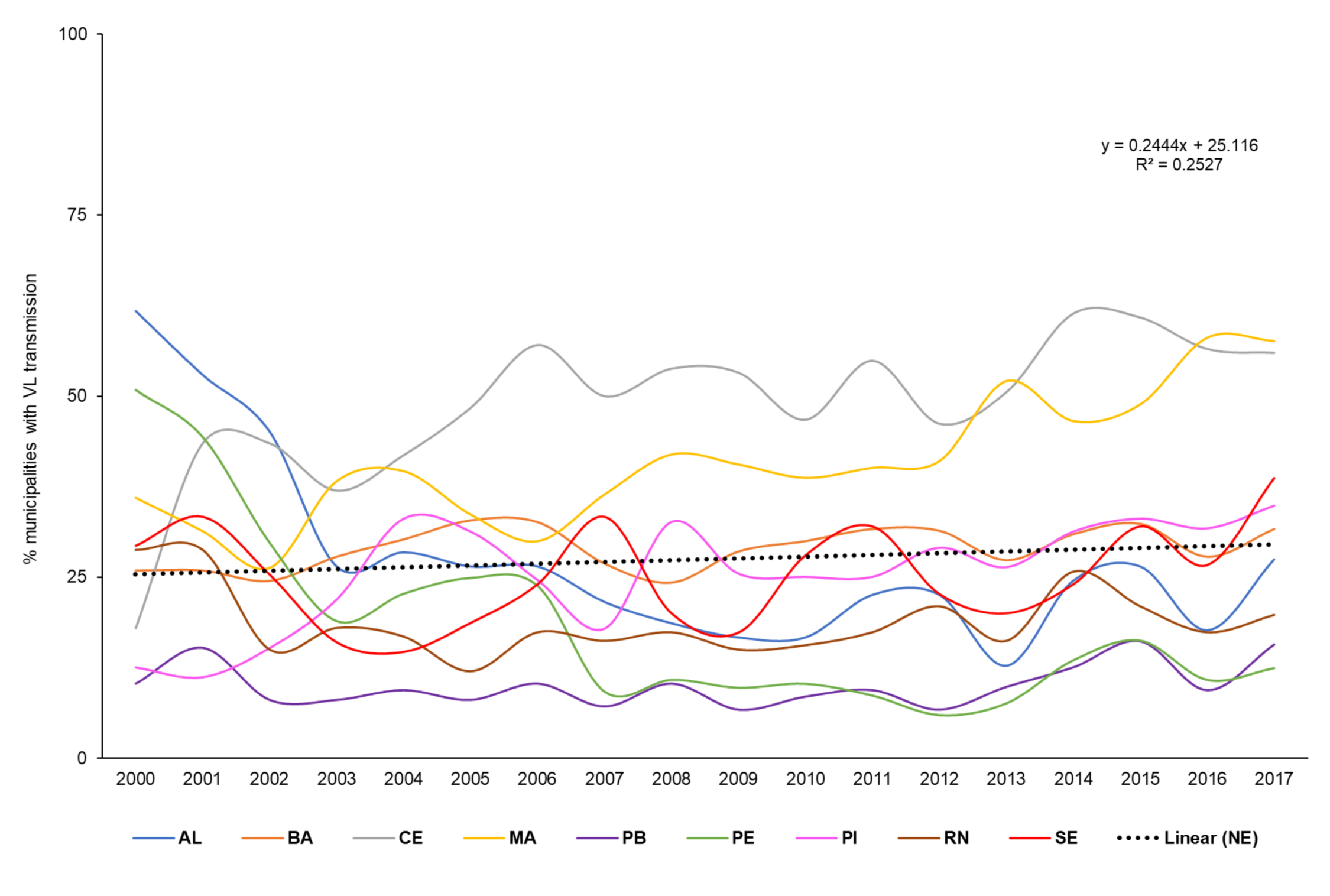

Supplement: S1 Fig — (TIF) [file pntd.0009006.s004.tif]
