# Supplementary material for: Space-time risk cluster of visceral leishmaniasis in Brazilian endemic region with high social vulnerability: An ecological time series study
Source: PLoS Negl Trop Dis. 2021 Jan 19;15(1):e0009006. doi: 10.1371/journal.pntd.0009006 (PMC7846114; doi:10.1371/journal.pntd.0009006)
Supplement: Attachment — Submitted filename: Responses.docx [file pntd.0009006.s005.docx]

Dear editor,

We would like to thank you for considering our manuscript. We performed all corrections as showed below:

-Please remove the Title Page file and ensure this is present as the first page of the manuscript file.

We merged the title page and the main document of manuscript.

- Please include this sentence in the “Financial Disclosure” field of the online submission form: “The funders had no role in study design, data collection and analysis, decision to publish, or preparation of the manuscript.” If this statement is not correct, you must describe the role of any sponsors or funders and modify the statement as needed. Please note that this statement is in addition to your current funding information; it should not replace it. The "Financial Disclosure" field is located in the “Additional Information” section of the online submission form. Please also ensure your Financial Disclosure is in complete sentences.

We added the sentence in the online form.

- We note that you have listed suggested reviewers that share the same affiliation as an author of your manuscript. Please remove these reviewers and substitute with different suggestions.

We excluded the reviewer and added two new reviewers as suggested.

-Our team has noticed that the maps/satellite images in Figures 1, 4, and 6 do not have any sources cited in their captions, and we cannot tell they are from copyrighted sources that are not publishable under our Open Access CC-BY License. You can find our policy here: [http://journals.plos.org/plosntds/s/licenses-and-copyright].

We request you please cite any programs and/or datasets used to create the maps in your Figure 1, 4, and 6 captions so that we can determine if they are publishable under our CC BY 4.0 license.

The information about the cartographic base (shapefile extension) is presented in the subsection “Data sources”, page 8, lines 183-185, and in the section of “Supporting information”, page 40, lines 755-757. The software used to produce the images is cited in the subsection “Softwares”, page 12, lines 271-273. Furthermore, we added this information in images captions, as requested.

- Additionally, please provide in your Ethics Statement:

--Your study involves the use of patient medical data. Please indicate in the ‘Ethics Statement’ that all data analyzed were anonymized.

We added the statement, as suggested.

We look forward to hearing good news from you.

Best wishes.

Tatiana R. de Moura
