# Supplementary material for: Space-time risk cluster of visceral leishmaniasis in Brazilian endemic region with high social vulnerability: An ecological time series study
Source: PLoS Negl Trop Dis. 2021 Jan 19;15(1):e0009006. doi: 10.1371/journal.pntd.0009006 (PMC7846114; doi:10.1371/journal.pntd.0009006)
Supplement: Attachment — Submitted filename: Letter.docx [file pntd.0009006.s006.docx]

**Federal University of Sergipe**

**Graduate Programme in Health Science**

Cláudio Batista Street, Sanatório District, Aracaju, Sergipe, Brazil

Phone: +55 79 3194-7202, E-mail: [tmoura.ufs@gmail.com](mailto:tmoura.ufs@gmail.com)

Aracaju, May 30, 2020

Sitara SR Ajjampur, MD, PhD

Guest Editor

PLOS Neglected Tropical Diseases

Genevieve Milon

Deputy Editor

PLOS Neglected Tropical Diseases

**Manuscript:** Space-time risk cluster of visceral leishmaniasis in Brazilian endemic region with high social vulnerability: an ecological time series study

PNTD-D-19-01930

Dear editors,

We would like to thank the reviewers for their excellent comments and suggestions for the manuscript. We performed a thorough review and revision of the full text, tables, and figures, following the reviewers’ recommendations and the journal guidelines. The feedback was instrumental to improving the quality of our submitted work. We provide in the next page a detailed list of responses. For a better understanding, we renumbered and organized the questions by each reviewer. We also show edits in the manuscript using track changes. We believe that this revised version is acceptable for publication in PLOS NTD.

**Answers to reviewers:**

**Reviewer #2:** *First of all I would like to say thanks for your contributions on this very sensitive issue visceral leishmaniosis in Brazil. The paper generally good but the author try to update all the necessary comments particularly in methodology section including variable classifications, definitions and statistical model formulations. Some editorial problem the author must modify as per the format of the journal including tables and figures. Updating recent studies on this area under discussion part and also modify the conclusion.*

**Question 1 -** *How can you calculate and measure the VL incidence rate in municipality level?*

**Answer 1 -** We thank the reviewer for pointing out it. We updated the manuscript to directly address this. Information can be found in lines 170-172.

**Question 2 -** *Study Variable classifications and definitions is not clear? (Response variable? Explanatory variables? Please summarize in tabular form.*

**Answer 2 –** Great suggestion. We updated the manuscript to now include a table (Table 1) that summarizes all variables with their respective classification, description, and type of analysis. Thanks.

**Question 3 -** *The methodological part is not clear for instance spatiotemporal cluster analysis try to elaborate on the Poisson probability discrete model, bivariate spatial analysis, try to rewrite explicitly including relevant dynamic spatiotemporal models. Try to put also the detail statistical model in short and precise manner.*

**Answer 3 -** We thank for the observation. We revised all the subsections of statistical methods and added respective citations, including substantial explanation about the analysis. Please see lines 236-248 and 251-274. Moreover, we added two new tables (Tables 5 and 6) to clarify the results of bivariate analysis.

**Question 4 -** *The result presented are good as descriptive but try to modify the tables 1 for base line characteristics if it is possible you have to present in readable manner and put in the previous page.*

**Answer 4 -** We agree with this excellent suggestion and have split the table into two. Table 2 now presents epidemiological characterization of all cases in regional level. Supplement 1 shows the same information, but in state level. I believe this will make it much clearer and readable. Thank you.

**Question 5 -** *In trend analysis you said it is stationary? How have you check test of stationarity? If so which test are used? If it is unit root what are you going to do with non-stationary trend /data? Please specify and define the test of Stationarity with its hypothesis testing procedures.*

**Answer 5 -** We thank the careful information about our statistical analysis. We have updated time the trends analysis section, clarified concepts and added supporting citations. Please see lines 199 through 211. Also, we changed the term “stationary” to “stable” to better reflect the state.

**Question 6 -** *In the result section you said there was a statistical significance? (How and when we say statistically significant) please interpret your results in relation to p-value.*

**Answer 6 –** Yes, we agree. We have updated the results section to now better present analyses and stats. The updates now better clarify the interpretation of data. Briefly, all increasing and decreasing trends had p-values<0.05 and did not include zero in their 95% CIs, such as the stable trends had p-values>0.05. Please see lines 199 through 211.

**Question 7 -** *Again put table 2 in previous page. Also put figure 4 on previous page.*

**Answer 7 –** Thank you for the advice. We made the suggested corrections.

**Question 8 -** *Moreover there are a lot of works are done on this area so try to include all recent and relevant works under discussion part including the dynamic spatiotemporal modeling in VL.*

**Answer 8 –** This is an excellent suggestion and the authors agree. Thank you very much. We have now included more references in the methods and discussion sections to better note the development of several studies in this area, especially in the Northeast region of Brazil. Please see lines 513-528.

**Question 9 -** *What are the limitations or gaps for this study? Please clearly state in precise manner. What are the key limitations of analysis? What is the contribution of this study in the area including public health relevance please update this part under discussion section?*

**Answer 9 -** We thank the careful information about our discussion. Several improvements were performed and have incorporated your suggestions into the discussion section. Please see lines 576-597.

**Reviewer #3:** *The authors conducted a novel investigation of the relationship between the incidence of a neglected tropical disease and social vulnerability in a region of Brazil with the highest historical prevalence and high degree of social inequalities. The study of health disparities is an important topic and health research priority. The study is thorough and with some improvements it could be considered for publication.*

**Question 1 -** *The authors provide a clear and concise overview of their study methodology, evident in their description of the SVI value range and its interpretation. Any mention of a statistical test should be met with a citation to help with reproducibility. Based on Figure 4C, the clustering does not appear to be circular, so what was the authors justification for each chosen scan statistic parameter?*

**Answer 1 -** We thank the reviewer for this important observation. To better support justification of our methodology, we included citations regarding SVI development. This index was elaborated by a governmental institution responsible for social statistics (*Instituto de Pesquisa Econômica Aplicada - IPEA -* <http://ivs.ipea.gov.br/index.php/pt/>) using data from the Brazilian *census*. Additionally, we cited previous works that applied SVI in their respective spatial analysis that demonstrated association between infectious diseases and social vulnerability (measured by SVI). In relation to employing the circular cluster parameter, we chose it based on similar previous studies that executed spatiotemporal analysis. The map generated from SaTScan is displayed as a circular cluster, but its edition is not available. So, the authors had to export the sheet results and all maps were evaluated in QGis, following methodologies adopted by previous studies.

**Question 2 -** *Reporting unadjusted statistics in Table 1 is important to report. Also reporting population adjusted statistics (e.g., using the population of Brazil an external reference group) would make comparing VL and other characteristics across the ten federative units/states. The authors include helpful detail to interpret the results, however, these details are best for the methods or discussion.*

**Answer 2 -** We thank the reviewer for this comment. Both reviewer #1 and #2 had similar suggestions regarding presentation of Table 1 data. Therefore, we split Table 1 into two parts to make the data clearer to readers. The purpose of this table is to describe the epidemiological characterization of all cases in the region.

**Question 3 -** *Figure 1 does not seem to add any detail to the paper and can be omitted.*

**Answer 3 –** Thank you for the suggestion and we have now excluded Figure 1.

**Question 4 -** *Based on Appendix 1, Appendix 2, and Appendix 3 the SVI are likely correlated. Presently, the study does neither assess this correlation, mention it in the discussion, or cite previous studies that may have done so already.*

**Answer 4 -** This is an excellent observation. We have now included more information about the methodology of SVI development, including appropriate citations. This index was elaborated by an institution linked to Ministry of Economy of Brazil and indicators extracted from the Brazilian census.

**Question 5 -** What characteristics of Maranhão and Ceará may explain why VL incidence is increasing?

**Answer 5 –** Great question. We expanded our discussion to include information not only about Maranhão and Ceará states, but also about Bahia, whose municipalities comprised 7 out of 12 spatiotemporal clusters. Please see lines 530-546. We believe this now better helps to explain the increased incidence of VL in this context.

**Question 6 -** The number of study limitations discussed are few and appear tucked away in paragraph starting on L521 with the study strengths. The ecological design of the study is not the only study limitation. Other examples the authors may want to consider are the potential limitations of using the SVIs, in general, or potential reporting issues of VL that may lead to uncertainty, or limitations of choices in statistical methodology.

**Answer 6 -** We thank the reviewer for the careful evaluation and critique of our discussion. It was very helpful in editing to enhance the readability within the discussion sections. Please see lines 577-597 for the updated text within the manuscript.

**Question 7 -** Besides the public policy angle, what insight about VL epidemiology or natural history does the investigation demonstrate? Or what further questions does the investigation raise and what future directions can this study be taken. For example, a

future direction could be to conduct spatial regression to further assess the relationship between VL and SVI beyond spatial- or spatiotemporal cluster detection.

**Answer 7 –** These are excellent questions and suggestions. Many thanks. We added this following information in lines 570-576: “Further evaluative studies are necessary to identify the critical points in the Brazilian VL program that have hindered the reduction in VL incidence and lethality in Northeast region. Moreover, it is urgent to develop researches with spatial regression and prospective spatiotemporal cluster analysis to identify the epidemiological and social determinants related to intense clustering of VL cases in states in the Northeastern sub-regions *sertão* and *meio-norte*.”

**Question 8 -** *Spell out “<“ when used in body of text*

**Answer 8 –** Corrections have been made. Thanks.

**Question 9 -** *L72 “Ecological and time series”, remove the ‘and’.*

**Answer 9 –** Thank you for catching the error. We have corrected.

**Question 10 -** *L253 typo.*

**Answer 10 –** Thanks. We corrected in the manuscript.

**Question 11 -** *L117 use of semicolon.*

**Answer 11 –** Item has been corrected.

**Question 12 -** *L102 typo.*

**Answer 12 –** Typo has been corrected. Thanks

**Question 13 -** *L321 the syntax in this section needs some tightening and double-check the formatting of all 95%CIs.*

**Answer 13 –** Thank you for pointing this out. We have cleaned up the text.

**Question 14 -** *L348 & L388 spell out “Fig” when in-line text*

**Answer 14 –** The word has been spelled out. Thanks.

**Question 15 -** *The sentence starting in L459 seems out of place.*

**Answer 7 –** We have cleaned up the sentence of line 459.

**Question 16 -** *Paragraph starting on L473 and L478 can be combined.*

**Answer 16 –** Great suggestion. Paragraphs have been combined.

**Question 17 -** *Table 2: Typo in a heading of a column.*

**Answer 17 –** We have corrected the error.

**Question 18 -** *Figure 4: suggestion to have one common compass rose and scale bar like in Figure 6.*

**Answer 18 –** Another great suggestion. Thank you. The figure has been updated based on your suggestion.

**Question 19 -** *Table 3: If indicated all clusters are statistically significant, then the authors may remove the p-value column.*

**Answer 19 -** We thank the reviewer for pointing it out. We agree with this suggestion. The column was deleted.

**Question 20 -** *Supplemental 2 Figure 1: Inclusion of an overall trend line combining all federative units/states would help with comparisons between federative units/states*

**Answer 20 -** We added a linear trend of percentage of municipalities with VL transmission in regional level, as well as the R²-value and the regression equation. Thank you for this great suggestion.

We again appreciate the thoroughness of each reviewer. The feedback has been instrumental to increasing the overall readability, quality, and strength of the manuscript. We were able to well address all reviewer comments and concerns and are looking forward to hearing good news in this revised manuscript.

Sincerely,

Tatiana Rodrigues de Moura, PhD

Associate Professor, Department of Morphology

Federal University of Sergipe, Brazil
