# Supplementary material for: Space-time risk cluster of visceral leishmaniasis in Brazilian endemic region with high social vulnerability: An ecological time series study
Source: PLoS Negl Trop Dis. 2021 Jan 19;15(1):e0009006. doi: 10.1371/journal.pntd.0009006 (PMC7846114; doi:10.1371/journal.pntd.0009006)
Supplement: Attachment — Submitted filename: Letter.docx [file pntd.0009006.s007.docx]

**Federal University of Sergipe**

**Graduate Programme in Health Science**

Cláudio Batista Street, Sanatório District, Aracaju, Sergipe, Brazil

Phone: +55 79 3194-7202, E-mail: [tmoura.ufs@gmail.com](mailto:tmoura.ufs@gmail.com)

Aracaju, Oct 22, 2020

Genevieve Milon

Deputy Editor

PLOS Neglected Tropical Diseases

**Manuscript:** Space-time risk cluster of visceral leishmaniasis in Brazilian endemic region with high social vulnerability: an ecological time series study

PNTD-D-19-01930R2

Dear editor,

We would like to thank the reviewer for his comments and suggestions. We performed a thorough review of the manuscript. The feedback was instrumental to improving the quality of our submitted work. We provide in the next page a detailed list of responses. We also show edits in the manuscript using track changes. We believe that this revised version is acceptable for publication in PLOS NTD.

**Answers to reviewer:**

**Reviewer #3:** L522: Great addition about data limitations. If there is evidence of underreporting at the health systems in Northeastern Brazil, then its citation would enhance your limitation section. If there is no evidence or study about health system underreporting then I would recommend adding “potential” before “underreporting of cases” and “missingness” replacing “the missing.” You could combine with the following sentence by removing “Maybe this situation occurs due the” with “possibly due to the.”

**Answer -** First of all, we would like to thank the reviewer for his considerations. We totally agree with this suggestion. We rewrote the sentence expressed at L522.

--------------------

**Editorial and Data Presentation Modifications?**

**Reviewer #3:** “global Moran’s index I” = “global Moran’s I index”
**Answer –** Thanks. We corrected this mistake in L219, L255, L340, L380, and L391.

L436: Typo “trough” = “through”’
**Answer –** Thanks. We corrected in the manuscript.

Table 2: “Miss data = “Missing data”
**Answer –** The word was replaced.

L369, L485: use of “whose” seems awkward here. Try “and” or removing
**Answer –** In L369 we switched “whose” for “and” and in L485 we removed it.

L456: Sentence “Similarly, in Brazil” is awkward, try “has been reported” at the end of the sentence.
**Answer –** We put the verb at the end of the sentence.

L465: Sentence “In Rio Grande do Norte…” is awkward, try removing “it was found” for “there is”
**Answer –** The expression was replaced.

L487: This is the first mention of Lutzomyia longipalpis so provide the full genus
**Answer –** We wrote the full genus.

L515: Suggestion to change “it is urgent to develop researches” to “there is a need to conduct investigations”

**Answer –** The expression was replaced according to suggestion.

S2 Appendix 1: Typo in column 2 row 3 “inclued” = “included” (it appears correctly in track-changed document)
**Answer –** Thanks. We corrected this mistake.

S2 Appendix 3: Typo “ie” = “i.e.,”

**Answer –** Thanks. We corrected this mistake.

We again appreciate the thoroughness of the reviewer. The feedback has been instrumental to increasing the overall readability, quality, and strength of the manuscript. We were able to well address all reviewer comments and concerns and are looking forward to hearing good news in this revised manuscript.

Sincerely,

Tatiana Rodrigues de Moura, PhD

Associate Professor, Department of Morphology

Federal University of Sergipe, Brazil
